# Genetic dissection of the roles of *β*-hydroxylases in carotenoid metabolism, photosynthesis, and plant growth in tetraploid wheat (*Triticum turgidum* L.)

**DOI:** 10.1007/s00122-023-04276-3

**Published:** 2023-01-19

**Authors:** Cody S. Bekkering, Shu Yu, Nina N. Isaka, Benjamin W. Sproul, Jorge Dubcovsky, Li Tian

**Affiliations:** Department of Plant Sciences, University of California, Mail Stop 3, Davis, CA 95616 USA

## Abstract

**Key message:**

Functional redundancy and subfunctionalization of *β*-hydroxylases in tetraploid wheat tissues open up opportunities for manipulation of carotenoid metabolism for trait improvement.

**Abstract:**

The genetic diversity provided by subgenome homoeologs in allopolyploid wheat can be leveraged for developing improved wheat varieties with modified chemical traits, including profiles of carotenoids, which play critical roles in photosynthesis, photoprotection, and growth regulation. Carotenoid profiles are greatly influenced by hydroxylation catalyzed by *β*-hydroxylases (HYDs). To genetically dissect the contribution of HYDs to carotenoid metabolism and wheat growth and yield, we isolated loss-of-function mutants of the two homoeologs of *HYD1* (*HYD-A1* and *HYD-B1*) and *HYD2* (*HYD-A2* and *HYD-B2*) from the sequenced ethyl methanesulfonate mutant population of the tetraploid wheat cultivar Kronos, and generated various mutant combinations. Although functional redundancy between HYD1 and HYD2 paralogs was observed in leaves, HYD1 homoeologs contributed more than HYD2 homoeologs to carotenoid *β*-ring hydroxylation in this tissue. By contrast, the HYD2 homoeologs functioned toward production of lutein, the major carotenoid in mature grains, whereas HYD1 homoeologs had a limited role. These results collectively suggested subfunctionalization of *HYD* genes and homoeologs in different tissues of tetraploid wheat. Despite reduced photoprotective responses observed in the triple *hyd-A1 hyd-B1 hyd-A2* and the quadruple *hyd-A1 hyd-B1 hyd-A2 hyd-B2* combinatorial mutants, comprehensive plant phenotyping analysis revealed that all mutants analyzed were comparable to the control for growth, yield, and fertility, except for a slight delay in anthesis and senescence as well as accelerated germination in the quadruple mutant. Overall, this research takes steps toward untangling the functions of HYDs in wheat and has implications for improving performance and consumer traits of this economically important global crop.

**Supplementary Information:**

The online version contains supplementary material available at 10.1007/s00122-023-04276-3.

## Introduction

Grown globally, wheat remains one of the most cultivated crops worldwide (Food and Agriculture Organization of the United Nations 2021) and supplies a substantial fraction of global protein and calorie needs. Tetraploid wheat (*Triticum turgidum*; AABB subgenome) is an allopolyploid formed by the hybridization of two diploid species: *T. urartu* (einkorn wheat; AA) and an unknown Sitopsis species of genus Aegilops (BB) about half a million years ago (Dvořák et al. 1993; Dvořák and Zhang 1990; Li et al. 2022). Subgenome homoeologs brought together by allopolyploidization have gradually changed in their gene expression profiles over the course of wheat adaptation to the environment and cultivation by humans (Ramírez-González et al. 2018). The presence of this set of subgenome homoeologs presents an opportunity for trait improvement in wheat by providing breeders more underlying genetic diversity to select upon or manipulate.

Carotenoid profile is one trait that can be fruitfully altered in wheat for improving plant performance and nutritional quality. Carotenoids can be divided into two groups: carotenes and xanthophylls, with the latter being oxygenated derivatives of carotenes. Carotenoid compounds collectively are important for processes central to plant physiology including photosynthesis, photoprotection, phytohormone biosynthesis, pigmentation, and stress response (Nisar et al. 2015). For instance, *β*-carotene is critical for photosynthesis in plants by serving as a pigment in photosynthetic reaction centers and light harvesting complexes (Qin et al. 2015). In addition, *β*-carotene acts as a precursor for apocarotenoid signaling molecules including the phytohormone strigolactone (Moreno et al. 2021). On the other hand, the xanthophyll cycle pigments zeaxanthin, antheraxanthin, and violaxanthin are central to the photoprotective response in higher plants (Demmig-Adams and Adams 1996), while the epoxy xanthophylls 9-*cis*-violaxanthin and 9′-*cis*-neoxanthin are further converted to the phytohormone abscisic acid (ABA) (Felemban et al. 2019). Carotenoid content in plants is also highly relevant for human health, as carotenoids containing unmodified *β*-ionone rings (*β*-rings; particularly *β*-carotene) are important precursors for vitamin A biosynthesis in humans.

**Fig. 1:**
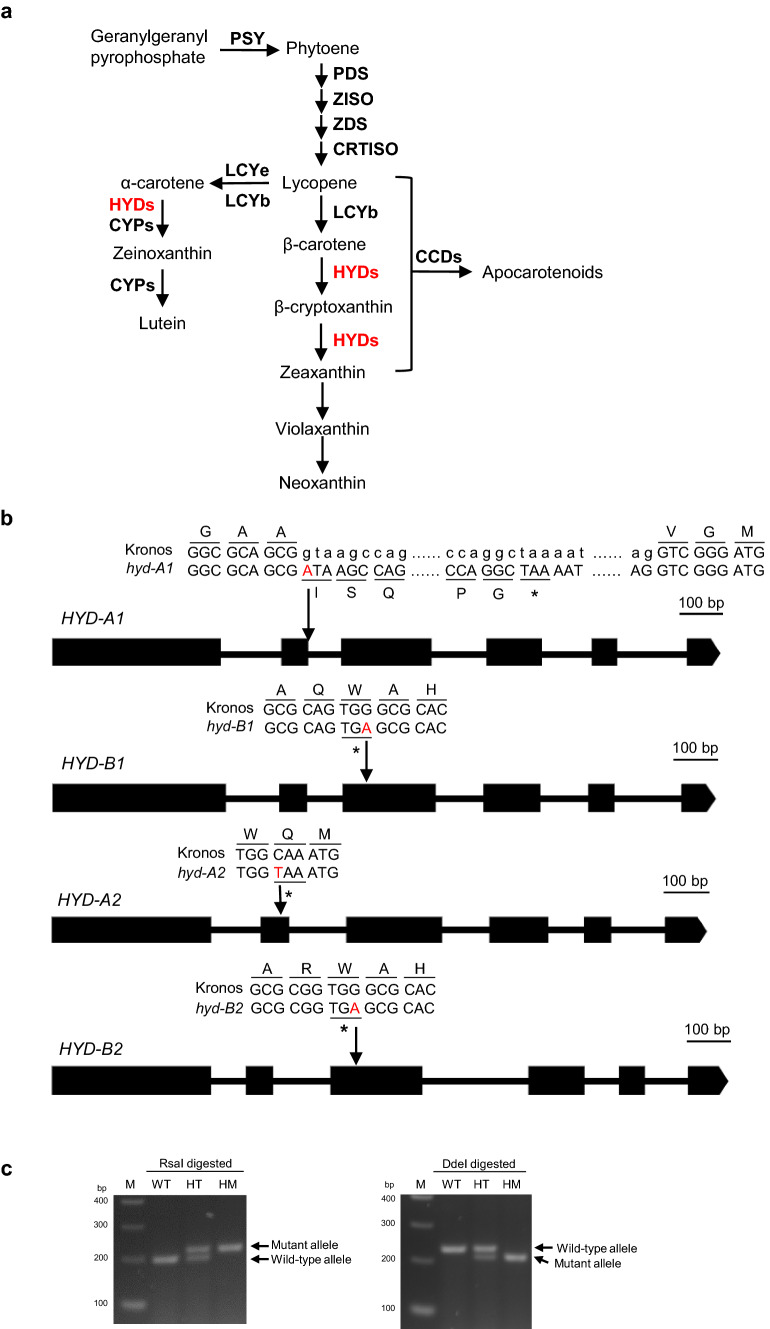
Identification of tetraploid wheat *hyd* mutants. **a** A simplified carotenoid biosynthetic pathway in wheat. Enzymes are indicated in bold. PSY, phytoene synthase; PDS, phytoene desaturase; ZISO, *ζ*-carotene isomerase; ZDS, *ζ*-carotene desaturase; CRTISO, carotenoid isomerase; LCYb, lycopene *β*-cyclase; LCYe, lycopene *ε*-cyclase; HYD, *β*-hydroxylase (non-heme di-iron type); CYP, cytochrome P450 type carotenoid hydroxylase; CCD, carotenoid cleavage dioxygenase. Enzyme activities blocked in the mutants are indicated in red. **b** Location of mutations within the *HYD* homoeologs in the tetraploid wheat mutants. The mutated nucleotides are highlighted in red. **c** Derived Cleaved Amplified Polymorphic Sequence (dCAPS) markers of *HYD-A1* and *HYD-B1* (WT: Wild type, HT: Heterozygous mutant, HM: Homozygous mutant). Markers of *HYD-A2* and *HYD-B2* were published previously (Yu et al. 2022)

Hydroxylation constitutes the key step in the conversion of carotenes to xanthophylls (Fig. 1a). The hydroxylation reactions are catalyzed by two groups of carotenoid hydroxylases in plants, including the cytochrome P450 type hydroxylases (CYPs) that act primarily on the *α*-carotene-branch (*β*, *ε*-branch) carotenoids and the non-heme di-iron type *β*-hydroxylases (HYDs) that prefer the *β*-carotene-branch (*β*,* β*-branch) carotenoids (Kim and DellaPenna 2006; Tian et al. 2003, 2004). Two HYD paralogs, HYD1 and HYD2, hydroxylate *β*-carotene to form zeaxanthin in many plants (Fig. 1a). Mutants in *HYD1* and *HYD2* have thus far been explored in *Arabidopsis thaliana* and a few other plant species, where both HYDs were found to play a role in converting *β*-carotene to xanthophyll cycle pigments (Diretto et al. 2007; Du et al. 2010; Kim et al. 2012; Li et al. 2010; Tian et al. 2003). Homoeologs of *HYD1* and *HYD2* have also been cloned from tetraploid and hexaploid wheat and biochemically characterized in an *E. coli* system (Qin et al. 2012). However, the individual and combinatorial contribution of each of these HYD homoeologs to carotenoid profiles and plant productivity in wheat remains to be elucidated.

To genetically dissect the contribution of HYDs to carotenoid metabolism, photosynthesis, and the growth and yield of wheat, we isolated loss-of-function mutants in each homoeolog of *HYD1* (*HYD-A1* and *HYD-B1*) and HYD2 (*HYD-A2* and *HYD-B2*) from a sequenced ethyl methanesulfonate (EMS) mutant population of tetraploid wheat cv. Kronos (Krasileva et al. 2017). Higher order mutant combinations including double, triple, and quadruple mutants of the *HYD1* and *HYD2* homoeologs were generated by crossing. The *hyd* mutant combinations and the control line were analyzed for their carotenoid contents in leaves and mature grains. Photosynthetic properties and plant growth parameters were also characterized for these combinatorial mutants. Finally, key components of yield were assessed under a semi-controlled (greenhouse) setting to infer the potential effect of these combined mutations on plant performance.

## Materials and methods

### Plant growth conditions

Wheat seeds were surface sterilized using a 1% (w/v) sodium hypochlorite and 0.1% (v/v) Triton X-100 solution for 35 min and rinsed at least 5 times with sterile distilled water. For genotyping and whole-plant phenotyping, as well as carotenoid and photosynthesis analyses, sterilized seeds were stored at 4 °C in dark for 3–4 days, then kept at room temperature under a constant light intensity of 120 μmol m^−2^ s^−1^ for 3 days. The seedlings were transferred to soil and grown in a temperature-controlled greenhouse. Light intensities in the greenhouse varied throughout the day but averaged around 400 μmol m^−2^ s^−1^. Supplemental lighting was supplied to plants to extend the photoperiod to a 16 h light and 8 h dark cycle. For greenhouse experiments, individual plants were grown in pots that were approximately 3 L in volume, with a top diameter of 16 cm, a bottom diameter of 13 cm, and a height of 18 cm. Plants were watered twice weekly with nutrient water (Table S1). Every fourth watering was done with distilled water to ensure that salts did not build up in the soil. Watering was stopped at the hard dough stage.

### Isolation of tetraploid wheat mutants, functional characterization of mutant homoeologs in *E. coli*, and generation of mutant combinations

A sequenced tetraploid wheat cv. Kronos EMS mutant population (Krasileva et al. 2017) was screened to identify mutants of the *HYD1* and *HYD2* homoeologs. Putative nonsense mutants for each *HYD* homoeolog were functionally characterized to verify loss-of-function. To clone the *hyd/HYD* homoeologs, leaves of the mutant and wild-type plants were collected and total RNA was extracted from 100 mg of leaf tissue using a GeneJET RNA Purification Kit (Thermo Fisher Scientific, Waltham, MA, USA); the total RNA was subsequently treated with DNase I (Thermo Fisher Scientific) to remove the residual genomic DNA in the sample. First-strand cDNA was synthesized using SuperScript™ III reverse transcriptase (Invitrogen, Carlsbad, CA, USA). The open reading frame of the wild-type *HYD* or the mutant *hyd* alleles was amplified using Phusion High-Fidelity DNA Polymerase (Thermo Fisher Scientific), cloned into the pENTR/D-TOPO vector (Invitrogen), and then recombined into pDEST17 (Invitrogen) using the Gateway LR Clonase II Enzyme Mix (Invitrogen). Primers used in the TOPO cloning and in the mutant screening are listed in Table S2.

The recombinant plasmids were transformed into *E. coli* JM109 cells that contain the plasmid pAC-BETA and produce *β*-carotene (Cunningham and Gantt 2007). Single colonies from each transformation were used to inoculate a 30-mL of Luria Bertani (LB) medium with 100 μg mL^−1^ ampicillin, 35 μg mL^−1^ chloramphenicol, and 1% glucose. The bacterial cultures were shaken at 220 rpm at 37 °C for 2 h and transferred to 25 °C to grow for another 16 h. The cells were then harvested, and the pellets resuspended in LB medium containing 100 μg mL^−1^ ampicillin, 35 μg mL^−1^ chloramphenicol, and 0.4 mM isopropyl *β*-d-1-thiogalactopyranoside (IPTG). The cells were grown at 37 °C for an additional 4 h with shaking at 220 rpm and then harvested. For carotenoid extraction, the bacterial pellets were resuspended in 300 μL of formaldehyde, followed by the addition of 300 μL of methanol, and then 600 μL of ethyl acetate. The mixture was centrifuged and the supernatant was transferred to a new tube. After phase separation by adding 400 μL of H_2_O, the upper ethyl acetate layer was transferred to a high-performance liquid chromatography (HPLC) vial, 10 μL of which was injected on a reverse phase HPLC and separated following a previously established gradient (Qin et al. 2012).

After verification of the non-functional *hyd* mutant alleles in *E. coli*, the loss-of-function tetraploid wheat mutant lines for *HYD-A1* (T4-2017), *HYD-B1* (T4-0641), *HYD-A2* (T4-0870), and *HYD-B2* (T4-4420) were designated as *hyd-A1*, *hyd-B1*, *hyd-A2*, and *hyd-B2*, respectively, and used for the following studies. These mutants were each backcrossed to the wild-type parental line Kronos two times to remove ~ 75% of background mutations caused by chemical mutagenesis. The homozygous, background-purified single mutants were intercrossed and genotyped to identify homozygous double mutants of the *HYD1* or *HYD2* homoeologs (*hyd-A1 hyd-B1* and *hyd-A2 hyd-B2*, respectively). The homozygous *hyd-A1 hyd-B1* and *hyd-A2 hyd-B2* mutants were crossed to produce quadruple mutant plants heterozygous for the *HYD-A1*, *HYD-B1*, *HYD-A2*, and *HYD-B2* loci. These plants were self-pollinated to produce a segregating population for identification of the homozygous single, double, triple, and quadruple mutants used in this study. The segregating progenies were genotyped for the *HYD-A1*, *HYD-B1*, *HYD-A2*, and *HYD-B2* loci to identify various mutant combinations using Cleaved Amplified Polymorphic Sequence (CAPS) and derived CAPS (dCAPS) markers generated for each of the four selected EMS mutations. Primers and restriction enzymes (New England Biolabs, Ipswich, MA) used for the dCAPS markers of *HYD-A1* and *HYD-B1* are shown in Table S2. The CAPS and dCAPS markers used for *HYD-A2* and *HYD-B2* were previously described (Yu et al. 2022). Restriction enzyme digestion was carried out for a minimum of 12 h at 37 °C before analysis by gel electrophoresis.

### Carotenoid and chlorophyll analysis

For carotenoid and chlorophyll analysis of leaves, the fourth leaf from the top was collected from the primary tiller of four-week-old wheat plants, flash-frozen in liquid nitrogen, ground into a fine powder in liquid nitrogen using a mortar and pestle, and stored at − 80 °C until analysis. Whole grains of wheat were frozen with liquid nitrogen and ground into a fine powder using a mortar and pestle. Carotenoid extraction from leaf (~ 50 mg) and whole grain (~ 300 mg) samples and HPLC analysis were conducted using established procedures (Qin et al. 2012; Yu et al. 2022). There were 5–7 biological replicates and 4 biological replicates for the leaf and grain carotenoid analysis, respectively.

### Measurement of photosynthetic parameters

Non-photochemical quenching (NPQ) induction and relaxation was measured using the LI-6400XT Portable Photosynthesis System (LI-COR Biosciences, Lincoln, NE, USA). Leaves from 12-week-old plants grown under greenhouse conditions were dark-adapted overnight by wrapping leaves completely in aluminum foil. The flag leaf of the primary tiller was used for measurement whenever possible. Flag leaves of other tillers were also used if the flag leaf of the primary tiller was beginning to senesce or was otherwise unsuitable. Between 4 and 6 biological replicates were measured for each genotype. Dark-adapted leaves were clamped into the fluorometer chamber and allowed for a further dark adaptation of 5 min prior to measurement. The leaves were exposed to an actinic light intensity of 1000 μmol m^−2^ s^−1^ for 6 min. After illumination for 6 min, actinic light was turned off for another 6 min to observe NPQ relaxation. NPQ was measured with a saturating pulse every 30 s in both the illuminated and dark state. The maximum quantum efficiency of Photosystem II (F_v_/F_m_) was determined with the first saturating pulse.

### Whole-plant phenotyping and pot assessment of yield related traits

Plant height was measured during the grain filling stage (~ 14 weeks old) as the distance from the soil line to the top of the highest spike (not including awns). Anthesis time for each plant was defined as the number of days from planting to the time when the first anthers became extruded. Senescence in wheat follows a characteristic pattern in spikes where green awn tissue browns gradually, and this pattern of browning proceeds down the spike. In this study, senescence was defined as the point where half of the spikes had no green tissue remaining between flag leaf ligule and the tip of the awns. The number of days from planting to this senescence was measured for the control plants and each of the mutant lines.

For pot yield assessment, plants were harvested after no green aerial tissues remained. Spike number, total spikelets per spike, and fertile spikelets per spike were determined manually for 4–6 plants of each genotype at harvest. Spikes were dried for 3 days at 37 °C and weighed after drying. Grains were counted and weighed after being manually separated from chaff. The chaff weight for each spike was computed as the difference between the dried spike mass and the total grain mass. Spike fertility index was defined as the number of grains per gram of spike chaff for each plant (Guo et al. 2018). Spikelet fertility rate was described as the percentage of fertile spikelets over total spikelets for each plant. Floret fertility was estimated by dividing the number of grains by the total number of spikelets produced by each plant. Late-emerging, infertile spikes were excluded from this analysis. Harvest index (ratio of grain mass to above-ground plant mass) was computed on a separate population consisting of 15 plants of each genotype. For this analysis, straw from individual plants was weighed after being dried for 3 days at 50 °C.

### Grain phenotyping, seed germination, and leaf water loss assays

Images of wheat grains were acquired with a flatbed document scanner (Epson Corporation, Nagano, Japan). Grains were spread across the scanner bed and placed with their crease facing downward. The glass bed of the scanner was covered with a transparency to prevent scratching and black paper was laid on top of the grains to improve contrast in the image. Scans were acquired at 300 dpi and stored as jpeg files. No color correction options were enabled during image acquisition. Grain parameters including length, width, perimeter, and greyscale values were measured from the images of 11–12 plants of each genotype using GrainScan (Whan et al. 2014).

Germination of seeds and loss of water in leaves were determined as previously described with modification (Yu et al. 2022). For the germination assay, seeds of the quadruple *hyd-A1 hyd-B1 hyd-A2 hyd-B2* mutant and the control were germinated under a constant light intensity of 120 μmol m^−2^ s^−1^ for 6 days. Five independent experiments were carried out, with 100 seeds of each genotype per experiment. For the water loss assay, 2-week-old *hyd-A1 hyd-B1 hyd-A2 hyd-B2* mutant and control plants (24 biological replicates of each genotype) were used.

### Root system phenotyping

Seminal root phenotypes were analyzed on 4-days-old wheat seedlings. Sterilized seeds were placed in transparent sheet protectors on a sheet of germination paper (Blue Blotter 76#; Anchor Paper Co., St. Paul, MN, USA) soaked with 40 mL of sterile distilled water. Germination paper was aligned with the bottom of the sheet protector; a single seed was placed in each sheet protector with its crease facing the germination paper (and with embryo facing downward) in the midpoint of the sheet and 2.5-cm from its top edge. Seeds were stratified at 4 °C for at least three days inside the sheet protectors. After stratification, sheets with seeds of different genotypes were placed randomly in a 3-ring binder and positioned vertically at room temperature (~ 22 °C) under long-day conditions (16 h/8 h, day/night) with a light intensity of 120 μmol m^−2^ s^−1^. Seeds that did not start germinating within 24 h were excluded from phenotyping. Developing seedlings (15 seedlings of each genotype) were imaged after 4 days using a document scanner (Epson). Prior to imaging, condensation was removed from the inside of the transparency using a Kimwipe (Kimberly-Clark, Irving, TX, USA). Images were acquired as 400 dpi TIF files.

Seminal root traits were assessed from images using RhizoVision Explorer (Seethepalli et al. 2021). A threshold level of 175 was used on inverted seedling images. Background objects less than 5 mm were automatically removed to eliminate the contribution of image artifacts to the analysis. Foreground objects (“holes”) shorter than 4 mm were also removed automatically. A root pruning threshold of 15 was used to prevent root hairs from being detected as lateral roots. Edge smoothing was not enabled. Before analysis in RhizoVision Explorer, seedling images were cropped using the GNU Image Manipulation Program (GIMP) to remove scanner and transparency edges as well as hypocotyls and grains. Root number was counted manually, and hypocotyl lengths were traced using ImageJ (Abramoff et al. 2004).

Root traits were also assessed on plants with more mature root systems at the three-leaf stage (~ 2.5 weeks old). Plants of each genotype (5–8 biological replicates) were grown in a liquid growth solution consisting of the same nutrient concentrations as used for the soil-grown plants (Table S1). These plants were also grown under similar, semi-controlled greenhouse conditions described above. Wheat seeds for this analysis were germinated in the germination sleeves as previously described for 4-days-old seedlings, transferred to cylindrical sponges roughly 2.5 cm in length and diameter, then mounted in a foam board floating in the nutrient solution. The tanks of nutrient solution had dimensions 24 cm × 24 cm × 16 cm, with each tank holding 4 plants that were spaced 5 cm apart. The nutrient solution was filled up to 3 cm from the top of the tank (approximately 9.2 L) and was changed twice weekly to ensure sufficient dissolved oxygen. Root length and shoot fresh weight were assessed at harvest. Root and shoot dry weight were measured after drying for 5 days at 37 °C. The root: shoot ratio, defined as the ratio of the dry mass of the root compared to the shoot, was also computed.

### Statistical analysis

When all genotypes were being evaluated, one-way analysis of variance (ANOVA) was performed followed by pairwise mean comparisons using Tukey’s Honestly Significant Difference (HSD) test. Student’s *t* test was used when only two genotypes were being assessed. All tests were carried out using JMP software (JMP Statistical Discovery LLC, Cary, NC, USA).

## Results

### *HYD1* and *HYD2* loss-of-function mutants were identified in tetraploid wheat and intercrossed to generate higher order mutant combinations

To investigate the contribution of HYDs to carotenoid metabolism and plant growth in wheat, a library of 1536 sequenced tetraploid wheat cv. Kronos EMS mutants (Krasileva et al. 2017) was screened and 63 *hyd-A1*, 60 *hyd-B1*, 50 *hyd-A2*, and 67 *hyd-B2* mutants were identified. Selected mutant line T4-2017 (*hyd-A1*) contains a G to A mutation at the exon–intron junction (donor splice site) of the second intron, leading to a 19-amino acid (AA) insertion followed by a premature stop codon, thus a net result of a 155-AA truncation of HYD-A1 (Fig. 1b). Selected mutant line T4-0641 (*hyd-B1*) contains a G to A mutation in the third exon, resulting in a premature stop codon (W158*), and a 160-AA truncation of HYD-B1 (Fig. 1b). The C to T mutation in the second exon of *HYD-A2* in selected mutant line T4-0870 (*hyd-A2*), and the G to A mutation in the third exon of *HYD-B2* in selected mutant line T4-4420 (*hyd-B2*) lead to premature stop codons in HYD-A2 (Q113*) and HYD-B2 (W149*), respectively (Fig. 1b). Each of these truncated HYD proteins lacks the conserved histidine residues shown to be essential for HYD activities (Bouvier et al. 1998). Indeed, hydroxylated products of *β*-carotene, i.e., *β*-cryptoxanthin or zeaxanthin, were not detected in *E. coli* cells co-expressing pAC-BETA (producing *β*-carotene) and the mutant alleles of *hyd-A1*, *hyd-B1*, *hyd-A2*, or *hyd-B2*, indicating that the proteins encoded by these truncated mutants are compromised for HYD function (Fig. S1).

To reduce the background mutations introduced by chemical mutagenesis, the homozygous *hyd-A1*, *hyd-B1*, *hyd-A2*, and *hyd-B2* mutants were backcrossed twice to the wild-type parental line Kronos. These backcrossed lines were then crossed to obtain the *hyd-A1 hyd-B1*, and *hyd-A2 hyd-B2* double mutants. In a segregating population generated by crossing *hyd-A1 hyd-B1* and *hyd-A2 hyd-B2* then selfing the progenies, homozygous single, double, triple, and quadruple mutants of the *HYD1* and *HYD2* homoeologs were identified with molecular markers (Fig. 1c). Henceforth, “single mutants” refers to the individual *hyd-A1*, *hyd-B1*, *hyd-A2*, and *hyd-B2* mutants; “double mutants” to *hyd-A1 hyd-B1* and *hyd-A2 hyd-B2*; “triple mutants” to *hyd-A1 hyd-A2 hyd-B2*, *hyd-B1 hyd-A2 hyd-B2*, *hyd-A1 hyd-B1 hyd-A2*, and *hyd-A1 hyd-B1 hyd-B2*; and “quadruple mutant” to *hyd-A1 hyd-B1 hyd-A2 hyd-B2*. “Control” refers to sister plants in the segregating population that contain homozygous wild-type *HYD-A1*, *HYD-B1*, *HYD-A2*, and *HYD-B2* alleles and a similar load of background mutations as those of the single, double, triple, and quadruple mutants.

### HYD homoeologs exhibited differential contributions to carotenoid profiles in leaves

To understand the role of HYD homoeologs in carotenoid accumulation in the leaf tissue, carotenoid composition and content in leaves of *hyd* mutants and control plants were analyzed and compared (Table 1). Neoxanthin and violaxanthin (*β*-carotene-derived xanthophylls; *β*-xanthophylls), lutein, and *β*-carotene represent the carotenoids that accumulate in leaves of the control and all mutant genotypes (Table 1). Of the single mutants analyzed, carotenoid profiles in *hyd-A2* and *hyd-B2* were comparable to the control, while violaxanthin was reduced by approximately 15% each in *hyd-A1* and *hyd-B1* (Table 1). Of the double mutants, *hyd-A2 hyd-B2* had carotenoid profiles similar to the control, while *hyd-A1 hyd-B1* showed an 8% reduction in neoxanthin, a 54% decrease in violaxanthin, an 11% increase in lutein, and a 6% increase in *β*-carotene relative to the control (Table 1). Total carotenoids of all the single and double mutants did not differ significantly (*P* < 0.05) from those of the control (Table 1).

**Table 1:** Carotenoid profiles in the leaf tissue of the *hyd* mutants and control plants. Carotenoid content is normalized by total chlorophyll content (mmol carotenoid mol^−1^ chlorophylls *a* + *b*)

Genotype	Neoxanthin	Violaxanthin	Lutein	*β*-carotene	Total carotenoids	*β*-xanthophylls	*β*,*ε*/*β*,*β*
Control	22.16 ± 0.50^a^	29.19 ± 1.19^a^	68.14 ± 1.96^d^	53.05 ± 1.34^de^	172.52 ± 3.24^a^	51.34 ± 1.39^a^	0.65 ± 0.01^f^
*hyd-A1*	22.05 ± 0.55^a^	24.67 ± 1.61^b^	70.49 ± 1.42^cd^	54.07 ± 0.87^cde^	171.28 ± 2.09^ab^	46.72 ± 1.27^b^	0.70 ± 0.02^e^
*hyd-B1*	21.93 ± 0.04^a^	24.91 ± 0.92^b^	69.73 ± 2.20^cd^	54.47 ± 0.95^cde^	171.04 ± 3.95^ab^	46.84 ± 1.15^b^	0.69 ± 0.01^e^
*hyd-A2*	21.81 ± 0.36^ab^	27.84 ± 1.24^ab^	67.20 ± 1.35^d^	54.01 ± 1.06^cde^	170.86 ± 3.17^ab^	49.65 ± 1.30^ab^	0.65 ± 0.01^f^
*hyd-B2*	21.99 ± 0.55^a^	27.46 ± 2.23^ab^	68.36 ± 1.88^d^	52.76 ± 2.00^e^	170.57 ± 6.13^ab^	49.45 ± 2.63^ab^	0.67 ± 0.02^ef^
*hyd-A1 hyd-B1*	20.35 ± 1.45^bc^	13.45 ± 1.22^d^	75.57 ± 3.01^b^	56.45 ± 1.91^bc^	165.82 ± 7.05^abcd^	33.80 ± 2.46^d^	0.84 ± 0.01^c^
*hyd-A2 hyd-B2*	22.13 ± 0.42^a^	26.55 ± 1.06^ab^	69.64 ± 1.41^cd^	54.07 ± 1.00^cde^	172.69 ± 3.18^a^	48.68 ± 1.22^ab^	0.68 ± 0.01^ef^
*hyd-A1 hyd-A2 hyd-B2*	21.48 ± 0.68^abc^	20.03 ± 1.11^c^	70.50 ± 1.57^cd^	53.98 ± 2.21^cde^	166.00 ± 4.34^abc^	41.51 ± 1.35^c^	0.74 ± 0.01^d^
*hyd-B1 hyd-A2 hyd-B2*	21.98 ± 0.72^a^	20.84 ± 1.50^c^	72.77 ± 1.73^bc^	54.68 ± 1.45^cde^	170.27 ± 2.70^ab^	42.82 ± 1.23^c^	0.75 ± 0.03^d^
*hyd-A1 hyd-B1 hyd-A2*	20.16 ± 0.78^c^	11.41 ± 1.29^e^	76.37 ± 2.64^b^	55.71 ± 1.65^cd^	163.66 ± 2.82^bcd^	31.57 ± 1.62^d^	0.88 ± 0.05^c^
*hyd-A1 hyd-B1 hyd-B2*	12.88 ± 1.27^d^	7.17 ± 0.39^f^	82.19 ± 2.82^a^	58.53 ± 0.93^ab^	160.77 ± 2.78^ cd^	20.05 ± 1.61^e^	1.05 ± 0.06^b^
*hyd-A1 hyd-B1 hyd-A2 hyd-B2*	6.86 ± 0.44^e^	5.16 ± 0.37^g^	85.46 ± 1.91^a^	60.73 ± 1.25^a^	158.21 ± 2.92^d^	12.02 ± 0.73^f^	1.18 ± 0.03^a^

The mean and standard deviation of 5–7 biological replicates are shown. *β*-xanthophylls include neoxanthin and violaxanthin. *β*,*ε*/* β*,* β* denotes the ratio of carotenoids in the *β*,*ε*-branch and the *β*,* β*-branch. Significantly different (*P* < 0.05) values within the same column are indicated with different superscript letters

Varying degrees of changes to carotenoid profiles were observed in triple and quadruple *hyd* mutants (Table 1). Two triple mutants, *hyd-A1 hyd-A2 hyd-B2* and *hyd-B1 hyd-A2 hyd-B2*, showed ~ 30% decreases in violaxanthin relative to the control. More fluctuations in carotenoid content were observed in the *hyd-A1 hyd-B1 hyd-A2* mutant, which had a 9% reduction in neoxanthin, a 61% reduction in violaxanthin, and a 12% increase in lutein. Stark differences in carotenoid profiles were observed in *hyd-A1 hyd-B1 hyd-B2*, which showed a 42% reduction in neoxanthin, a 75% reduction in violaxanthin, a 21% increase in lutein, and a 10% increase in *β*-carotene (Table 1). While* hyd-A1 hyd-A2 hyd-B2* and *hyd-B1 hyd-A2 hyd-B2* did not differ from the control in total carotenoids, *hyd-A1 hyd-B1 hyd-A2* and *hyd-A1 hyd-B1 hyd-B2* exhibited a 5% and 7% loss in total carotenoids, respectively. The results from the triple mutants suggested that HYD1 homoeologs play a more important role than HYD2 homoeologs in the biosynthesis of *β*-xanthophylls in leaves (Table 1).

The quadruple mutant, *hyd-A1 hyd-B1 hyd-A2 hyd-B2,* had the most drastically reduced neoxanthin and violaxanthin levels relative to the control (69% and 82% reductions, respectively; Table 1). *β*-carotene and lutein accumulated in higher levels (14% and 25% increases, respectively) in leaves of *hyd-A1 hyd-B1 hyd-A2 hyd-B2* than those of the control. However, total carotenoids decreased by only 8% in the quadruple mutant when compared to the control (Table 1).

### NPQ induction and relaxation was mostly compromised in the quadruple mutant

**Fig. 2:**
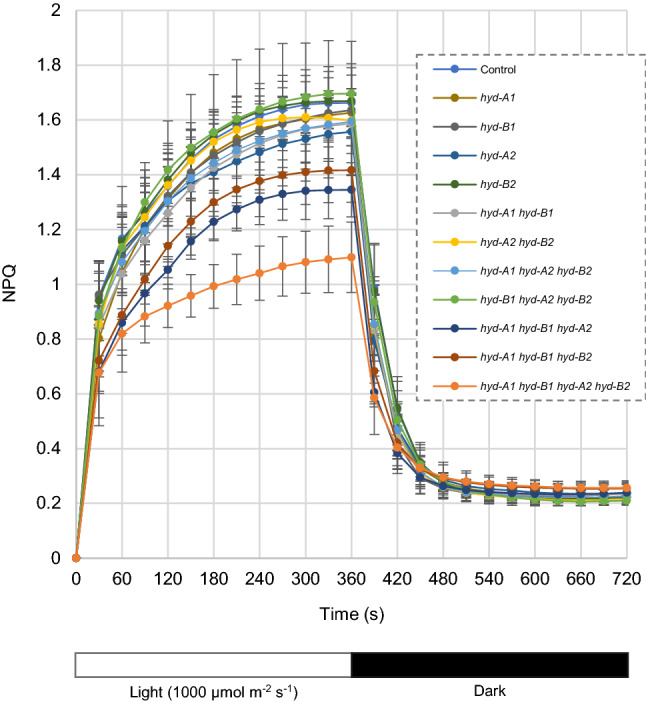
Induction and relaxation of non-photochemical quenching (NPQ) in dark-adapted leaves of *hyd* mutants and control plants. Mean values and standard deviations of 4–6 biological replicates are shown. The statistical analysis of the NPQ values during light induction is shown in Table S3

To investigate how changes in carotenoid profiles may affect photosynthesis in the *hyd* mutants relative to the control, chlorophyll content, maximum quantum efficiency of Photosystem II (F_v_/F_m_), and NPQ induction and relaxation were determined (Tables 2 and S3; Fig. 2). In the single mutant, double mutant, and triple mutant lines, no significant difference (*P* < 0.05) was observed in chlorophyll *a* (Chl *a*), chlorophyll *b* (Chl *b*), total chlorophylls (Chl *a* + *b*), or chlorophyll *a*/*b* ratio (Chl *a/b*) relative to the control (Table 2). However, in the quadruple mutant *hyd-A1 hyd-B1 hyd-A2 hyd-B2,* a 23% decrease in Chl *b* and a 16% decrease in Chl *a* were observed relative to the control alongside of an increased Chl *a/b* ratio, reflecting a larger proportional decrease in Chl *b* (Table 2).

**Table 2:** Chlorophyll content (μmol g^−1^ fresh tissue) and photosynthetic parameters of the *hyd* mutants and control plants

Genotype	Chl *b*	Chl *a*	Chl *a* + *b*	Chl *a*/*b*	Fv/Fm
Control	1.14 ± 0.04^a^	3.02 ± 0.15^a^	4.16 ± 0.18^a^	2.65 ± 0.09^a^	0.801 ± 0.004^a^
*hyd-A1*	1.11 ± 0.08^a^	2.92 ± 0.17^a^	4.02 ± 0.24^a^	2.63 ± 0.07^a^	0.796 ± 0.019^a^
*hyd-B1*	1.12 ± 0.04^a^	2.94 ± 0.15^a^	4.06 ± 0.19^a^	2.61 ± 0.06^a^	0.801 ± 0.013^a^
*hyd-A2*	1.11 ± 0.06^a^	2.99 ± 0.21^a^	4.09 ± 0.26^a^	2.70 ± 0.03^a^	0.801 ± 0.012^a^
*hyd-B2*	1.09 ± 0.06^a^	2.92 ± 0.15^a^	4.01 ± 0.21^a^	2.66 ± 0.05^a^	0.796 ± 0.012^a^
*hyd-A1 hyd-B1*	1.12 ± 0.05^a^	2.94 ± 0.13^a^	4.07 ± 0.18^a^	2.63 ± 0.09^a^	0.793 ± 0.006^a^
*hyd-A2 hyd-B2*	1.14 ± 0.05^a^	3.01 ± 0.14^a^	4.15 ± 0.19^a^	2.64 ± 0.06^a^	0.799 ± 0.012^a^
*hyd-A1 hyd-A2 hyd-B2*	1.11 ± 0.07^a^	2.93 ± 0.19^a^	4.04 ± 0.25^a^	2.65 ± 0.07^a^	0.795 ± 0.004^a^
*hyd-B1 hyd-A2 hyd-B2*	1.12 ± 0.10^a^	2.92 ± 0.21^a^	4.04 ± 0.30^a^	2.61 ± 0.11^a^	0.783 ± 0.01^a^
*hyd-A1 hyd-B1 hyd-A2*	1.02 ± 0.13^a^	2.72 ± 0.27^ab^	3.76 ± 0.39^ab^	2.61 ± 0.12^a^	0.802 ± 0.01^a^
*hyd-A1 hyd-B1 hyd-B2*	1.02 ± 0.06^a^	2.70 ± 0.16^ab^	3.72 ± 0.20^ab^	2.66 ± 0.08^a^	0.806 ± 0.016^a^
*hyd-A1 hyd-B1 hyd-A2 hyd-B2*	0.88 ± 0.04^b^	2.53 ± 0.16^b^	3.41 ± 0.19^b^	2.87 ± 0.09^b^	0.793 ± 0.01^a^

The mean and standard deviation of 5–7 biological replicates are shown. Significantly different (*P* < 0.05) values within the same column are indicated with different superscript letters. Chl, chlorophyll

Despite differences in leaf chlorophyll and carotenoid content observed in some of the mutants, the Fv/Fm values did not differ significantly (*P* < 0.05) between any of the mutant lines (including the quadruple mutant) and the control (Table 2). Maximum NPQ as well as the rates of NPQ induction clustered together for the control, the single and double mutants, as well as two triple mutants: *hyd-A1 hyd-A2 hyd-B2* and *hyd-B1 hyd-A2 hyd-B2* (Fig. 2; Table S3). However, NPQ induction was significantly (*P* < 0.05) reduced in *hyd-A1 hyd-B1 hyd-A2* and marginally reduced in *hyd-A1 hyd-B1 hyd-B2* (Table S3). The quadruple mutant *hyd-A1 hyd-B1 hyd-A2 hyd-B2* displayed slower NPQ induction and lower max NPQ (a ~ 34% reduction) than the control (Fig. 2; Table S3). On the other hand, NPQ relaxation rates were similar for all lines assessed, and all lines relaxed to a stable level within five minutes of removing actinic light (Fig. 2).

### HYD homoeologs contributed differently to carotenoid profiles in mature grains than in leaves

To determine the impact of *hyd* mutations on grain carotenoid production, carotenoid composition and content in mature grains were determined in all mutant genotypes and the control. Lutein and *β*-carotene were the two carotenoid compounds accumulated in mature grains (Table 3). Grains of the single mutants and the control contained similar levels of lutein, β-carotene, and total carotenoids (Table 3). However, significantly lower accumulation of lutein (34–45% reduction) was detected in each of the lines with mutations in both homoeologs of *HYD2*, including the double mutant *hyd-A2 hyd-B2*, the triple mutants of *hyd-A1 hyd-A2 hyd-B2* and *hyd-B1 hyd-A2 hyd-B2*, and the quadruple mutant *hyd-A1 hyd-B1 hyd-A2 hyd-B2*, relative to the control (Table 3). In addition, *β*-carotene was increased by 100–194% in these lines compared to the control. In contrast, the double and triple mutant lines with mutations in both homoeologs of *HYD1* (*hyd-A1 hyd-B1*, *hyd-A1 hyd-B1 hyd-A2*, and *hyd-A1 hyd-B1 hyd-B2*) had similar levels of lutein and *β*-carotene to the control, suggesting that the HYD1 homoeologs, unlike the HYD2 homoeologs, do not play a major role in the production of these carotenoids in mature tetraploid wheat grains (Table 3).

**Table 3:** Carotenoid profiles in mature whole grains of the *hyd* mutants and control plants. Carotenoid content is normalized by the weight of grain flour (nmol carotenoid g^−1^ flour)

Genotype	Lutein	*β*-carotene	Total carotenoids	*β*,*ε*/*β*,*β*
Control	7.44 ± 1.07^a^	0.49 ± 0.10^d^	7.94 ± 1.04^a^	15.74 ± 4.75^ab^
*hyd-A1*	6.32 ± 0.74^abcd^	0.49 ± 0.07^d^	6.81 ± 0.77^ab^	12.98 ± 1.62^abc^
*hyd-B1*	6.65 ± 0.67^abc^	0.40 ± 0.05^d^	7.05 ± 0.70^ab^	16.63 ± 1.72^a^
*hyd-A2*	6.74 ± 0.65^abc^	0.65 ± 0.13^bcd^	7.39 ± 0.75^ab^	10.55 ± 1.73^bc^
*hyd-B2*	6.78 ± 0.86^ab^	0.61 ± 0.24^cd^	7.39 ± 1.07^ab^	11.97 ± 3.18^abc^
*hyd-A1 hyd-B1*	6.80 ± 1.46^ab^	0.56 ± 0.12^d^	7.36 ± 1.49^ab^	12.46 ± 2.83^abc^
*hyd-A2 hyd-B2*	4.11 ± 0.52^d^	1.05 ± 0.23^ab^	5.16 ± 0.70^b^	4.03 ± 0.64^d^
*hyd-A1 hyd-A2 hyd-B2*	4.50 ± 0.32^bcd^	1.08 ± 0.27^ab^	5.58 ± 0.58^ab^	4.33 ± 0.85^d^
*hyd-B1 hyd-A2 hyd-B2*	4.42 ± 0.38^cd^	1.00 ± 0.14^bc^	5.42 ± 0.51^ab^	4.43 ± 0.30^d^
*hyd-A1 hyd-B1 hyd-A2*	6.25 ± 1.26^abcd^	0.83 ± 0.22^bcd^	7.07 ± 1.45^ab^	7.70 ± 1.16^cd^
*hyd-A1 hyd-B1 hyd-B2*	6.58 ± 1.55^abc^	0.54 ± 0.04^d^	7.11 ± 1.56^ab^	12.24 ± 2.97^abc^
*hyd-A1 hyd-B1 hyd-A2 hyd-B2*	4.93 ± 0.88^bcd^	1.44 ± 0.24^a^	6.37 ± 1.10^ab^	3.43 ± 0.27^d^

The mean and standard deviation of 4 biological replicates are shown. *β*,*ε*/* β*,* β* denotes the ratio of carotenoids in the *β*, *ε*-branch and the *β*,* β*-branch. Significantly different (*P* < 0.05) values within the same column are indicated with different superscript letters

### Comprehensive plant phenotyping revealed that the *hyd* mutants are mostly comparable to the control in growth and pot yield related traits except for a slight developmental delay in the quadruple mutant

**Fig. 3:**
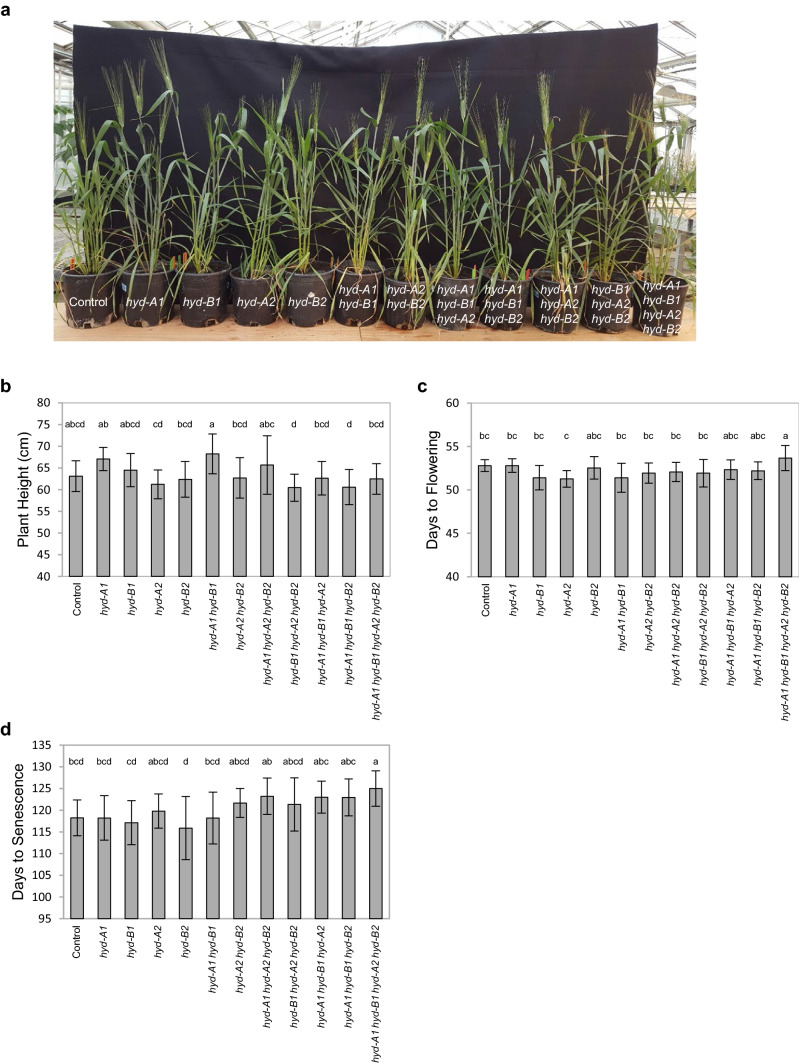
Plant growth phenotyping analysis of *hyd* mutants and control plants. **a** Image of *hyd* mutants and control plants during the grain filling stage **b** Plant heights **c.** Days to anthesis **d** Days to senescence. Mean values and standard deviations are displayed from 15 plants for each genotype

**Fig. 4:**
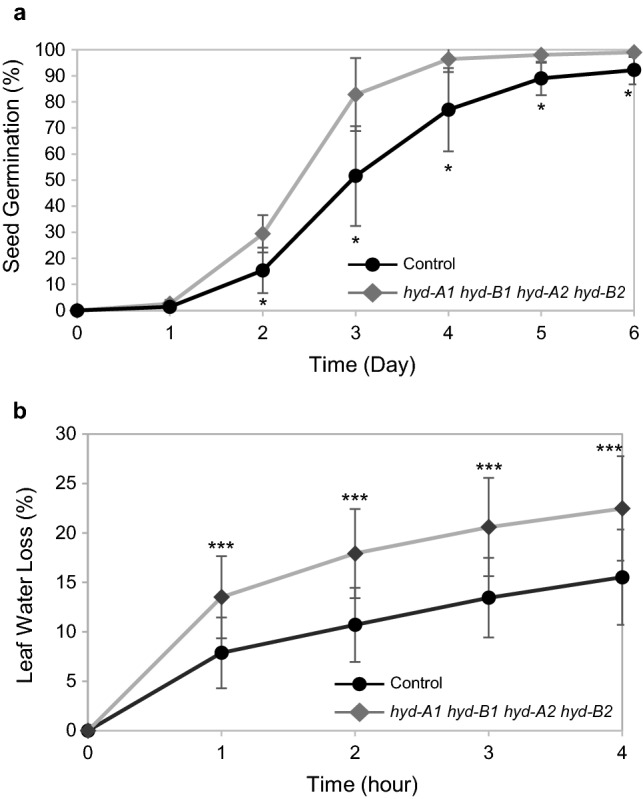
Rates of seed germination and leaf water loss of the quadruple mutant (*hyd-A1 hyd-B1 hyd-A2 hyd-B2*) and the control plants. **a** Cumulative germination rate of 100 seeds over time at ambient room temperature (~ 24 °C). Values are means ± standard deviations of 5 independent experiments each consisting of 100 seeds. *, *P* < 0.05. **b** Leaf water loss. Leaves of two-week-old plants were detached and left at ambient room temperature (~ 24 °C). They were weighed initially (0 h) and in 1-h intervals. Values shown are means ± standard deviations (*n* = 24). ***, *P* < 0.001

**Fig. 5:**
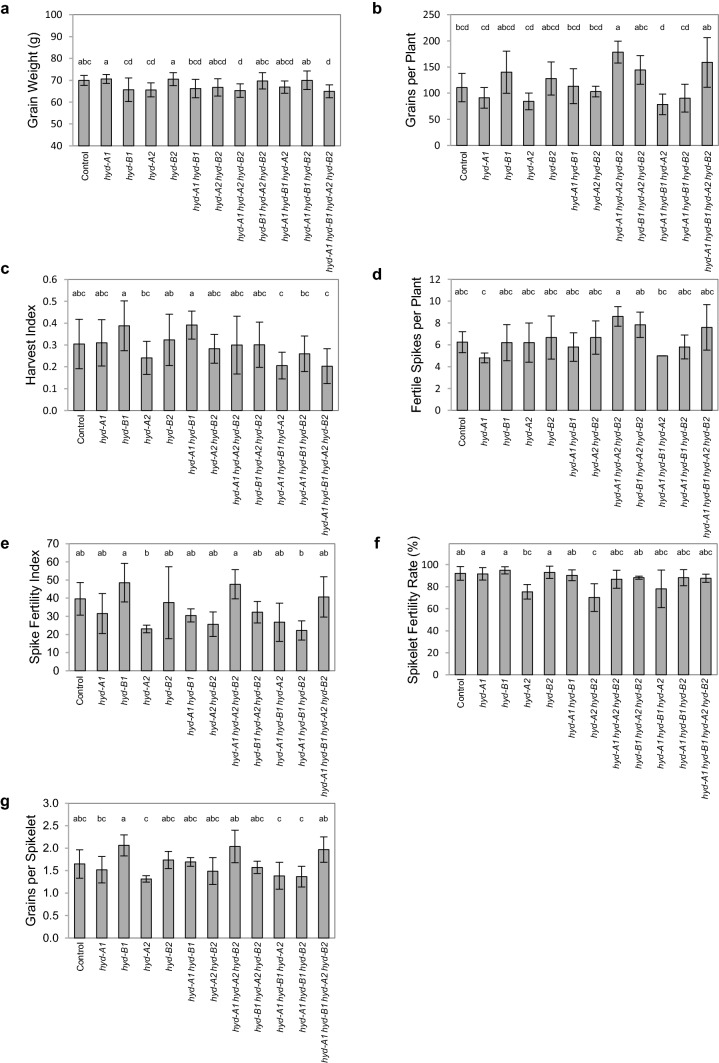
Yield analysis of the *hyd* mutant and control plants grown in a semi-controlled (greenhouse) environment. **a** Average weight of individual grains per plant **b** Grains per plant **c** Harvest index **d** Spikes per plant **e** Spike fertility index (grain number per gram of spike chaff) **f** Spikelet fertility rate (% of fertile spikelets as a portion of total spikelets per plant) **g** Grains per spikelet (grain number divided by total spikelets per plant). The data shown in **a** and **c** were collected from 15 plants for each genotype, whereas the data presented in **b** and **d–g** were collected from fertile spikes of 4–6 plants for each genotype

To explore the effect of *hyd* mutations on the physiology and productivity of tetraploid wheat plants, plants grown in a semi-controlled environment (greenhouse) were examined for their growth and pot yield traits (Figs. 3, 4, 5). Plant height did not differ significantly (*P* < 0.05) among the *hyd* mutants and the control at both flowering stage and grain filling stage (Fig. 3a, b). While there were no obvious differences in the overall growth and development of the single, double, and triple mutant lines relative to the control, the quadruple mutant *hyd-A1 hyd-B1* *hyd-A2* *hyd-B2* displayed a slight, but noticeable, delay in anthesis and senescence—with anthers extruding one day later and plants reaching senescence seven days later on average (Fig. 3c, d). It is worth noting that this delay in anthesis and senescence in the quadruple mutant was consistent across several independent experiments. When compared to the control, the quadruple mutant exhibited accelerated seed germination (Fig. 4a) and a faster water loss in detached leaves at room temperature (Fig. 4b). However, we did not observe any abnormal premature seed germination for the quadruple mutants when grown in the greenhouse (Fig. S2).

To evaluate the effects of *hyd* mutations on seedling establishment, coleoptile lengths and seminal root traits were assessed on 4-day-old seedlings to survey possible changes to root system architectures (Table 4). The seminal root traits included number of seminal roots, total root length, root convex area, and maximum depth. Despite occasional small differences between mutant genotypes, phenotypic values for seedlings of the *hyd* mutants and the control were similar (Table 4). The consistency of root growth across the genotypes was also observed in plants at the 3-leaf stage grown in liquid nutrient solution, where no differences (*P* < 0.05) were observed in the root length, dry weight, or root:shoot ratio (Table S4) across any of the mutant genotypes examined relative to the control.

**Table 4:** Phenotyping analysis of germinating 4-day-old seedlings of the *hyd* mutants and control plants

	Coleoptile length (cm)	Seminal root count	Total seminal root length (mm)	Convex area (mm^2^)	Seminal root max depth (mm)
Control	7.71 ± 1.79^ab^	5.00 ± 0.88^abc^	413.87 ± 99.73^b^	3726.67 ± 1700.95^ab^	111.33 ± 24.77^a^
*hyd-A1*	7.23 ± 1.34^ab^	4.83 ± 0.83^abc^	429.36 ± 131.43^b^	3733.09 ± 2292.02^ab^	101.04 ± 16.02^a^
*hyd-B1*	6.96 ± 1.91^b^	4.53 ± 0.92^bc^	400.67 ± 105.38^b^	3115.04 ± 1067.65^b^	101.23 ± 16.20^a^
*hyd-A2*	7.10 ± 2.20^b^	5.15 ± 0.38^abc^	491.78 ± 121.54^ab^	5506.90 ± 2822.81^ab^	116.93 ± 13.08^a^
*hyd-B2*	6.98 ± 1.68^b^	5.54 ± 0.52^a^	456.73 ± 82.78^b^	3370.71 ± 1874.31^ab^	109.08 ± 14.80^a^
*hyd-A1 hyd-B1*	7.23 ± 1.67^ab^	5.07 ± 0.96^ab^	450.75 ± 141.39^ab^	3933.00 ± 2048.83^ab^	107.94 ± 21.39^a^
*hyd-A2 hyd-B2*	6.84 ± 2.12^b^	4.33 ± 0.90^c^	419.01 ± 92.75^b^	3624.50 ± 1997.34^ab^	111.38 ± 11.38^a^
*hyd-A1 hyd-A2 hyd-B2*	9.03 ± 2.08^a^	5.64 ± 0.63^a^	590.54 ± 141.52^a^	5320.96 ± 2330.50^a^	108.97 ± 21.74^a^
*hyd-B1 hyd-A2 hyd-B2*	7.56 ± 1.80^ab^	4.93 ± 0.47^abc^	452.00 ± 120.82^b^	4325.05 ± 2628.68^ab^	107.69 ± 16.49^a^
*hyd-A1 hyd-B1 hyd-A2*	7.58 ± 1.91^ab^	4.86 ± 0.66^abc^	445.41 ± 118.44^b^	3380.18 ± 1051.19^ab^	116.51 ± 23.61^a^
*hyd-A1 hyd-B1 hyd-B2*	8.14 ± 1.83^ab^	5.29 ± 0.61^ab^	484.00 ± 89.57^ab^	4028.36 ± 1843.37^ab^	118.24 ± 18.46^a^
*hyd-A1 hyd-B1 hyd-A2 hyd-B2*	7.99 ± 1.48^ab^	5.00 ± 0.82^abc^	433.44 ± 98.37^b^	2939.58 ± 1270.10^ab^	98.52 ± 16.84^a^

The mean and standard deviation of 15 seedlings are shown. Significantly different (*P* < 0.05) values within the same column are indicated with different superscript letters

To understand the impact of *hyd* mutations on plant productivity, several pot yield components were examined in the *hyd* mutants and the control. These traits included grain morphology, grain weight and yield, harvest index (grain mass/total above-ground plant mass), fertile spike numbers per plant, spike fertility index (grain numbers/spike chaff mass), spikelet fertility rate (fertile spikelets/total spikelets), and grains per spikelet (Figs. 5 and S2; Table S5). No substantial differences in grain size, dimensions or weight were observed for any of the mutants relative to the control (Fig. 5a; Table S5). Grains from the examined lines were visually similar, though grains from the quadruple mutant line were slightly paler than those of the control and the single and double mutant lines, supported by the moderately increased greyscale values from grains of the quadruple mutant (Fig. S2). For yield related traits including grains per plant, harvest index, and fertile spikes per plant, as well as the fertility of spikes and spikelets, phenotypic values of *hyd* mutant lines were generally comparable to those of the control (Fig. 5b–g).

## Discussion

In this study, we generated and comprehensively characterized a collection of loss-of-function, combinatorial mutants of *HYD* homoeologs, which enabled us to untangle the contribution of each HYD homoeolog to carotenoid metabolism as well as the physiology and productivity of tetraploid wheat plants. The resemblance of carotenoid profiles and phenotypes of the single mutants and some of the double mutants to the control suggests that functional redundancy exists across the HYD homoeologs. However, characterization of the different *hyd* mutant combinations also revealed the subfunctionalization of HYD1 and HYD2 in carotenoid metabolism in leaf and mature grain tissues (Tables 1 and 3).

Violaxanthin and neoxanthin (*β*-xanthophylls) in the *β*,* β*-branch and lutein in the *β*,*ε*-branch are the three xanthophylls produced and accumulated in leaves (Fig. 1; Table 1). Knocking out either or both *HYD2* homoeologs did not affect carotenoid profiles in leaves, suggesting that the presence of HYD1 homoeologs can compensate for the lack of HYD2 activities in *hyd-A2*, *hyd-B2*, and *hyd-A2 hyd-B2*. On the other hand, eliminating the activity of a single HYD1 homoeolog impacted vioxanthin, and lines compromised in both HYD-A1 and HYD-B1 displayed reduced accumulation of neoxanthin and vioxanthin. These results suggest that homoeologs of HYD1 likely contribute the major carotenoid hydroxylase activity for *β*-xanthophyll biosynthesis in leaves, and that the functional loss of individual HYD1 homoeologs in the leaf tissue can only be partially compensated for by the other HYD1 homoeolog and the HYD2 homoeologs (Table 1). However, HYD2 homoeologs likely still function in *β*-xanthophyll biosynthesis in leaves, as the level of *β*-xanthophylls was higher in the *hyd-A1 hyd-B1*, *hyd-A1 hyd-B1 hyd-A2*, and *hyd-A1 hyd-B1 hyd-B2* mutants than in the quadruple mutant where the activities of both HYD2 homoeologs were eliminated in the absence of HYD1 (Table 1). Furthermore, the more severely impacted *β*-xanthophyll accumulation in *hyd-A1 hyd-B1 hyd-B2* (~ 40% reduction) relative to *hyd-A1 hyd-B1 hyd-A2* suggests a relatively more important role of HYD-B2 than HYD-A2 in *β*-xanthophyll biosynthesis in leaves (Table 1).

Despite having all four *HYD* homoeologs knocked out, the quadruple mutant synthesized a severely reduced but detectable amount of *β*-xanthophylls in the leaf tissue (Table 1). This result suggests that the CYP-type carotenoid hydroxylases can also add hydroxyl groups to *β*-carotene and *β*-cryptoxanthin (3-hydroxy-*β*-carotene) to produce zeaxanthin (3,3′-dihydroxy-*β*-carotene), which is subsequently converted to violaxanthin and neoxanthin; such activities of CYP carotenoid hydroxylases were also suggested by the analysis of the *Arabidopsis thaliana cyp97a3* mutant (Kim and DellaPenna 2006). The largely reduced *β*-xanthophylls in *hyd-A1 hyd-B1 hyd-B2* and the quadruple mutant *hyd-A1 hyd-B1 hyd-A2 hyd-B2* were accompanied by an increase in the levels of *β*-carotene (*β*,* β*-branch; due to a block of the HYD activities) and lutein (*β*,*ε*-branch) (Fig. 1; Table 1)*.* The latter could be the result of a shift in carbon flow from the *β*,* β*-branch to the *β*,*ε*-branch of the carotenoid pathway when the HYD activities are compromised (Table 1) (Tian et al. 2003). Moreover, the increase in lutein in these mutants, particularly in the quadruple mutant, suggests that CYP carotenoid hydroxylases can catalyze the hydroxylation on both the *ε*- and *β*-rings of *α*-carotene to form lutein in these mutants.

Another interesting finding from this study is the tissue-specific role of HYD2 homoeologs in tetraploid wheat grains. Only *β*-carotene and lutein had detectable accumulation in mature grains of tetraploid wheat (Table 3), while previous studies using developing grain tissues showed accumulation of *β*-xanthophylls in immature grains (Qin et al. 2012). Knocking out both *HYD-A2* and *HYD-B2* homoeologs led to 45% reduced lutein in grains of *hyd-A2 hyd-B2*, indicating that the HYD2 homoeologs in grains contribute to the hydroxylation of the *β*-ring of *α*-carotene to form zeinoxanthin (3-hydroxy-*α*-carotene), which is subsequently converted to lutein (Table 3). Interestingly, removing the activity of all four HYD homoeologs in the quadruple mutant did not lead to a further reduction of lutein relative to *hyd-A2 hyd-B2*, suggesting that the HYD1 homoeologs do not play a role in lutein production in mature tetraploid wheat grains (Table 3). Taken together, the carotenoid analysis results in leaves and grains collectively suggested subfunctionalization of the HYD homoeologs in these tissues.

The function of HYD paralogs (HYD1 and HYD2) in vegetative and reproductive tissues was also studied in other plant species, such as maize (Berman et al. 2017). *Zm*BCH2 (*β*-hydroxylase 2) was shown to be the major HYD activity for the conversion of *β*-carotene to zeaxanthin in maize endosperm through RNAi-mediated silencing of *ZmBCH2* (Berman et al. 2017). Two candidate transcription factors, *Zm*PBF and *Zm*GAMYB, were suggested for the regulation of *ZmBCH2* expression in developing grains by interacting with the proximal P-box and the AACA motif in the *ZmBCH2* promoter, respectively (Jin et al. 2019). Differential expression of *HYD* paralogs was previously revealed in wheat leaf and grain tissues (Qin et al. 2016). Our genetic analysis in this study further corroborated the role of HYD2 in carotenoid hydroxylation in tetraploid wheat grains (Table 3). However, the *cis*- and *trans*-elements responsible for transcriptional regulation of carotenoid hydroxylation remain uncharacterized in wheat and the regulatory mechanism for tissue-specific *HYD* expression patterns requires further investigation.

Earlier investigations have shown that the growth and yield of plants could be affected when carotenoid profiles are modified. For example, overexpression of lycopene *β*-cyclase (*LCYb*) genes from plant and bacterial sources in transgenic and transplastomic tomato plants changed their carotenoid profiles and resulted in rebalancing of biomass partitioning in vegetative and reproductive tissues (Mi et al. 2022). In other studies, manipulation of the xanthophyll cycle carotenoids in tobacco and *A. thaliana* led to enhanced and compromised plant growth and biomass production under fluctuating light conditions, respectively (Garcia-Molina and Leister 2020; Kromdijk et al. 2016). Considering the important roles of carotenoids in plant biomass production, the mutant population generated in this study was also leveraged to unravel the contributions of HYD homoeologs in tetraploid wheat plant growth and yield. Although reduced photoprotective responses were observed in *hyd-A1 hyd-B1 hyd-A2* and *hyd-A1 hyd-B1 hyd-A2 hyd-B2* (Fig. 2; Table S3), all the mutant plants were generally comparable to the control for growth (Fig. 3; Table 4; Table S4), suggesting a great growth plasticity for tetraploid wheat plants with modified carotenoid profiles under a semi-controlled condition. Notably, however, the quadruple mutant plants showed a slightly delayed anthesis and senescence than the control and other mutant genotypes. These results suggest that a severe perturbation in carotenoid metabolism, such as those observed in the quadruple *hyd* mutant, can affect the developmental program of the mutant plants. The ABA-related traits including seed germination and leaf water loss were modified in the quadruple mutant relative to the control, suggesting that the developmental delay could be partially contributed by a change in ABA regulation or its crosstalk with other plant hormones (Fig. 4). The underlying mechanism for the slightly delayed anthesis and senescence when both HYD1 and HYD2 were knocked out remains to be elucidated. Nevertheless, the delayed anthesis and senescence in the quadruple mutant did not affect pot yield or yield component traits such as the fertility of spikelets and spikes (Fig. 5).

Overall, this genetic analysis in tetraploid wheat unraveled redundancy and, more importantly, subfunctionalization of HYD homoeologs, which enable specific manipulation of carotenoid metabolism in one tissue without compromising carotenoid production and function in other tissues. This information is critical for ongoing attempts to increase *β*-carotene content in wheat grains. High levels of plasticity were also observed for growth phenotypes and yield in the *hyd* combinatorial mutants when grown in a semi-controlled environment. To further examine the extent of this phenotypic plasticity, the *hyd* mutants and the control plants are currently being assessed for agronomic traits in field experiments—supplying further insights into the performance of the different mutant combinations under multiple concurrent environmental stresses.

## Supplementary Information

Below is the link to the electronic supplementary material.

Supplementary file1 (DOCX 38 KB)

Supplementary file2 (PDF 338 KB)

## Data Availability

All data pertinent to the reported work are provided in the manuscript or supplemental online materials.

## Abbreviations

AA: Amino acid
ABA: Abscisic acid
ANOVA: Analysis of variance
CAPS: Cleaved amplified polymorphic sequence
Chl: Chlorophyll
CYP: Cytochrome P450 type hydroxylase
dCAPS: Derived cleaved amplified polymorphic sequence
GIMP: GNU image manipulation program
HPLC: High-performance liquid chromatography
HSD: Honestly significant difference
HYD: *β*-hydroxylase
IPTG: Isopropyl *β*-d-1-thiogalactopyranoside
LB: Luria Bertani
NPQ: Non-photochemical quenching
