# Supplementary material for: Genetic dissection of the roles of β-hydroxylases in carotenoid metabolism, photosynthesis, and plant growth in tetraploid wheat (Triticum turgidum L.)
Source: Theor Appl Genet. 2023 Jan 19;136(1):8. doi: 10.1007/s00122-023-04276-3 (PMC9852137; doi:10.1007/s00122-023-04276-3)
Supplement: Supplementary file 1 — Supplementary file1 (DOCX 38 KB) [file 122_2023_4276_MOESM1_ESM.docx]

**Supplementary Figure Legends**

**Fig. S1** Functional characterization of wild-type *HYD* and mutant *hyd* gene alleles using an *E. coli* system accumulating β-carotene. Wild-type HYD-A1, HYD-B1, HYD-A2, and HYD-B2 (**a, c, e, g**) were used as positive controls for β-hydroxylase activity. Carotenoid profiles of β-carotene-accumulating *E. coli* cells transformed with the mutant *hyd* alleles are shown in **b, d, f, h**. Truncated β-glucuronidase (tGUS) was used as a negative control for β-hydroxylase activity (**i**). β-car: β-carotene; zeax: zeaxanthin; β-cry: β-cryptoxanthin

**Fig. S2** Representative grains from the *hyd* mutants and control plants. **a.** An image of grains. **b.** Mean greyscale channel values for grains from plants of the control and the *hyd* mutants (n = 11-12). Statistically significant differences (*P* < 0.05) between groups are noted with different letters

**Supplementary Tables**

**Table S1** Elemental composition of the nutrient solution supplied to plants under greenhouse conditions. This solution was pH adjusted, with a final pH ranging from 5.3-6.0.

| Nutrient | Concentration (mg L^-1^) |
| --- | --- |
| NH_4_ | 10 |
| NO_3_ | 138 |
| P | 50 |
| K | 200 |
| Ca | 175 |
| Mg | 55 |
| S | 120 |
| Fe | 2.5 |
| Cu | 0.02 |
| B | 0.5 |
| Mn | 0.5 |
| Mo | 0.01 |
| Zn | 0.05 |
| Ni | 0.02 |

**Table S2** Primers used for mutant screening, cloning, and plant genotyping. Restriction enzymes used with CAPS and dCAPS markers are shown. Mismatched nucleotides in dCAPS markers are underlined

| Application | Gene | Forward (5’🡪3’) | Reverse (5’🡪3’) | Restriction Enzyme |
| --- | --- | --- | --- | --- |
| Mutant  Screening | *HYD-A1* | CTCCGTCCCTGTCCATTT | ATAGCAACCACCAAAAAGTAAC |  |
|  | *HYD-B1* | ATCCTTACCGTTAGTGAGTGGCGAATT | CTGCCTGATCATTAGCTCTG |  |
|  | *HYD-A2* | CCTTCCAATTTCCGCTCCTTTAC | GGGTAAGTCGGTGGCATATAAATA |  |
|  | *HYD-B2* | CCTCGGCATCACCTCCAT | GCAAGGCAGGTAAGAGCTGG |  |
| Cloning | *HYD-A1* | CACCATGGCCGCTGGTCTCTCGGG | ATAGCAACCACCAAAAAGTAAC |  |
|  | *HYD-B1* | CACCATGGCCGCTGGTCTCTCGGG | TCACAAGGCTCTTCTAGCTGCA |  |
|  | *HYD-A2* | CACCATGGCCGTCGCGAGGCTGGTG | GGGTAAGTCGGTGGCATATAAATA |  |
|  | *HYD-B2* | CACCATGGCCGTCGCGAGGCTGGTG | GCAAGGCAGGTAAGAGCTGG |  |
| Genotyping  (dCAPS) | *HYD-A1* | TGTCCAGCCTCGGCGTCACCTC | TTCTACCAAGGTGCCGCTGGCGTA | RsaI |
|  | *HYD-B1* | GGGATGGAGTTCTGGGCGCACTG | GGGTTTGCGAGTGGAGTGGATG | DdeI |
|  | *HYD-A2* | TCTACTACCGATTCGCCTGT | GTGTAAGTAAAGTCAGTGCTCACC | MseI |
| Genotyping  (CAPS) | *HYD-B2* | CCTCGGCATCACCTCCAT | GGACCAGAGTGGTTGATCTGCTG | HphI |

**Table S3** Mean values for non-photochemical quenching (NPQ) during light induction with standard deviations (n = 4-6). Groups with different superscript letters within each row are statistically significantly different (*P* < 0.05)

|  | Control | *hyd-A1* | *hyd-B1* | *hyd-A2* | *hyd-B2* | *hyd-A1*  *hyd-B1* | *hyd-A2*  *hyd-B2* | *hyd-A1 hyd-A2 hyd-B2* | *hyd-B1 hyd-A2 hyd-B2* | *hyd-A1 hyd-B1 hyd-A2* | *hyd-A1*  *hyd-B1*  *hyd-B2* | *hyd-A1 hyd-B1 hyd-A2 hyd-B2* |
| --- | --- | --- | --- | --- | --- | --- | --- | --- | --- | --- | --- | --- |
| 30 s | 0.96 ± 0.08^a^ | 0.80 ± 0.19^a^ | 0.96 ± 0.24^a^ | 0.88 ± 0.19^a^ | 0.94 ± 0.18^a^ | 0.85 ± 0.03^a^ | 0.86 ± 0.17^a^ | 0.89 ± 0.12^a^ | 0.89 ± 0.12^a^ | 0.68 ± 0.12^a^ | 0.72 ± 0.14^a^ | 0.68 ± 0.15^a^ |
| 60 s | 1.17 ± 0.08^a^ | 1.04 ± 0.14^a^ | 1.11 ± 0.22^a^ | 1.12 ± 0.22^a^ | 1.16 ± 0.21^a^ | 1.04 ± 0.09^a^ | 1.13 ± 0.10^a^ | 1.08 ± 0.15^a^ | 1.13 ± 0.14^a^ | 0.86 ± 0.16^a^ | 0.89 ± 0.14^a^ | 0.82 ± 0.13^a^ |
| 90 s | 1.26 ± 0.1^a^ | 1.20 ± 0.10^ab^ | 1.21 ± 0.20^ab^ | 1.21 ± 0.17^ab^ | 1.27 ± 0.22^a^ | 1.16 ± 0.15^ab^ | 1.24 ± 0.04^a^ | 1.19 ± 0.17^ab^ | 1.30 ± 0.13^a^ | 0.97 ± 0.16^ab^ | 1.02 ± 0.18^ab^ | 0.88 ± 0.12^b^ |
| 120 s | 1.36 ± 0.1^ab^ | 1.31 ± 0.08^ab^ | 1.32 ± 0.19^ab^ | 1.30 ± 0.13^ab^ | 1.38 ± 0.21^ab^ | 1.26 ± 0.18^abc^ | 1.36 ± 0.03^ab^ | 1.30 ± 0.16^ab^ | 1.42 ± 0.11^a^ | 1.05 ± 0.17^bc^ | 1.14 ± 0.20^abc^ | 0.92 ± 0.10^c^ |
| 150 s | 1.46 ± 0.08^ab^ | 1.41 ± 0.08^ab^ | 1.41 ± 0.18^ab^ | 1.37 ± 0.11^ab^ | 1.48 ± 0.2^ab^ | 1.35 ± 0.19^ab^ | 1.45 ± 0.05^ab^ | 1.39 ± 0.14^ab^ | 1.50 ± 0.09^a^ | 1.16 ± 0.16^bc^ | 1.23 ± 0.20^abc^ | 0.96 ± 0.08^c^ |
| 180 s | 1.53 ± 0.07^a^ | 1.48 ± 0.08^a^ | 1.47 ± 0.16^a^ | 1.41 ± 0.10^a^ | 1.55 ± 0.19^a^ | 1.43 ± 0.21^a^ | 1.52 ± 0.06^a^ | 1.44 ± 0.14^a^ | 1.55 ± 0.08^a^ | 1.23 ± 0.15^ab^ | 1.30 ± 0.18^ab^ | 0.99 ± 0.08^b^ |
| 210 s | 1.58 ± 0.07^ab^ | 1.53 ± 0.09^ab^ | 1.52 ± 0.15^ab^ | 1.45 ± 0.08^ab^ | 1.60 ± 0.19^ab^ | 1.47 ± 0.22^ab^ | 1.56 ± 0.08^ab^ | 1.49 ± 0.14^ab^ | 1.60 ± 0.07^a^ | 1.27 ± 0.14^bc^ | 1.35 ± 0.16^ab^ | 1.02 ± 0.09^c^ |
| 240 s | 1.61 ± 0.07^ab^ | 1.57 ± 0.10^ab^ | 1.56 ± 0.14^ab^ | 1.48 ± 0.08^ab^ | 1.63 ± 0.19^a^ | 1.52 ± 0.22^ab^ | 1.59 ± 0.09^ab^ | 1.52 ± 0.13^ab^ | 1.64 ± 0.06^a^ | 1.31 ± 0.12^bc^ | 1.38 ± 0.14^ab^ | 1.04 ± 0.10^c^ |
| 270 s | 1.64 ± 0.08^a^ | 1.59 ± 0.11^ab^ | 1.59 ± 0.14^ab^ | 1.51 ± 0.08^ab^ | 1.65 ± 0.19^a^ | 1.55 ± 0.21^ab^ | 1.61 ± 0.10^ab^ | 1.55 ± 0.12^ab^ | 1.67 ± 0.06^a^ | 1.33 ± 0.10^bc^ | 1.40 ± 0.12^ab^ | 1.07 ± 0.11^c^ |
| 300 s | 1.66 ± 0.08^a^ | 1.60 ± 0.11^ab^ | 1.61 ± 0.12^ab^ | 1.53 ± 0.08^ab^ | 1.66 ± 0.19^a^ | 1.57 ± 0.20^ab^ | 1.61 ± 0.11^ab^ | 1.57 ± 0.12^ab^ | 1.68 ± 0.05^a^ | 1.34 ± 0.09^bc^ | 1.41 ± 0.11^ab^ | 1.08 ± 0.12^c^ |
| 330s | 1.66 ± 0.09^a^ | 1.62 ± 0.12^ab^ | 1.62 ± 0.12^ab^ | 1.55 ± 0.09^ab^ | 1.67 ± 0.19^a^ | 1.58 ± 0.19^ab^ | 1.61 ± 0.10^ab^ | 1.58 ± 0.12^ab^ | 1.70 ± 0.06^a^ | 1.34 ± 0.09^bc^ | 1.42 ± 0.14^ab^ | 1.09 ± 0.13^c^ |
| 360s | 1.66 ± 0.1^a^ | 1.63 ± 0.13^ab^ | 1.64 ± 0.11^ab^ | 1.56 ± 0.09^ab^ | 1.67 ± 0.20^a^ | 1.59 ± 0.19^ab^ | 1.60 ± 0.10^ab^ | 1.59 ± 0.12^ab^ | 1.70 ± 0.06^a^ | 1.34 ± 0.09^bc^ | 1.42 ± 0.16^ab^ | 1.10 ± 0.14^c^ |

**Table S4** Growth measurements from *hyd* mutant and control plants at the 3-leaf stage (approximately 2.5 weeks old) grown in a liquid nutrient solution. The mean and standard deviation of 5-8 biological replicates are shown. Significantly different (*P* < 0.05) values within the same column are indicated with different superscript letters

|  | Root depth (cm) | Root dry weight (mg) | Shoot fresh weight (mg) | Shoot dry weight (mg) | Root:Shoot ratio |
| --- | --- | --- | --- | --- | --- |
| Control | 19.3 ± 3.0^a^ | 19.7 ± 7.0^a^ | 234.3 ± 70.0^a^ | 36.8 ± 8.6^a^ | 0.54 ± 0.13^a^ |
| *hyd-A1* | 18.3 ± 3.7^a^ | 19.1 ± 6.4^a^ | 216.3 ± 64.1^a^ | 34.3 ± 8.4^a^ | 0.56 ± 0.14^a^ |
| *hyd-B1* | 15.0 ± 3.9^a^ | 15.8 ± 5.6^a^ | 208.3 ± 67.7^a^ | 34.6 ± 9.8^a^ | 0.45 ± 0.06^a^ |
| *hyd-A2* | 16.0 ± 3.1^a^ | 16.8 ± 5.0^a^ | 182.5 ± 59.0^a^ | 33.1 ± 11.0^a^ | 0.51 ± 0.04^a^ |
| *hyd-B2* | 15.3 ± 2.4^a^ | 16.0 ± 3.0^a^ | 178.0 ± 34.2^a^ | 29.9 ± 3.7^a^ | 0.54 ± 0.09^a^ |
| *hyd-A1 hyd-B1* | 19.0 ± 2.2^a^ | 16.6 ± 3.2^a^ | 190.0 ± 43.4^a^ | 32.6 ± 5.9^a^ | 0.52 ± 0.13^a^ |
| *hyd-A2 hyd-B2* | 17.2 ± 2.6^a^ | 16.4 ± 4.1^a^ | 195.0 ± 47.8^a^ | 34.8 ± 6.8^a^ | 0.47 ± 0.08^a^ |
| *hyd-A1 hyd-A2 hyd-B2* | 18.9 ± 3.7^a^ | 18.9 ± 7.3^a^ | 191.3 ± 107.2^a^ | 33.1 ± 14.4^a^ | 0.59 ± 0.15^a^ |
| *hyd-B1 hyd-A2 hyd-B2* | 16.6 ± 3.5^a^ | 16.1 ± 4.4^a^ | 176.3 ± 47.5^a^ | 32.7 ± 6.2^a^ | 0.53 ± 0.27^a^ |
| *hyd-A1 hyd-B1 hyd-A2* | 18.9 ± 2.8^a^ | 19.2 ± 3.6^a^ | 228.8 ± 85.4^a^ | 38.9 ± 12.2^a^ | 0.52 ± 0.11^a^ |
| *hyd-A1 hyd-B1 hyd-B2* | 15.9 ± 6.1^a^ | 17.6 ± 7.5^a^ | 215.7 ± 67.5^a^ | 38.4 ± 8.3^a^ | 0.44 ± 0.11^a^ |
| *hyd-A1 hyd-B1 hyd-A2 hyd-B2* | 18.3 ± 2.9^a^ | 18.4 ± 4.1^a^ | 193.8 ± 44.4^a^ | 37.5 ± 8.0^a^ | 0.50 ± 0.08^a^ |

**Table S5** Mean dimensions and sizes of grains of *hyd* mutants and control plants with standard deviations (n = 11-12). Groups with different superscript letters within each row are statistically significantly different (*P* < 0.05)

|  | Seed Size (mm^2^) | Length (mm) | Width (mm) | Perimeter (mm) |
| --- | --- | --- | --- | --- |
| Control | 22.3 ± 1.2^ab^ | 7.8 ± 0.3^ab^ | 7.8 ± 0.3^ab^ | 23.3 ± 0.7^ab^ |
| *hyd-A1* | 21.9 ± 0.7^ab^ | 7.7 ± 0.2^ab^ | 7.7 ± 0.2^ab^ | 23.1 ± 0.4^ab^ |
| *hyd-B1* | 22.2 ± 0.8^ab^ | 7.8 ± 0.1^ab^ | 7.8 ± 0.1^ab^ | 23.4 ± 0.4^ab^ |
| *hyd-A2* | 21.6 ± 0.7^ab^ | 7.5 ± 0.1^b^ | 7.5 ± 0.1^b^ | 22.8 ± 0.3^b^ |
| *hyd-B2* | 22.7 ± 1.2^a^ | 7.8 ± 0.1^a^ | 7.8 ± 0.1^a^ | 23.6 ± 0.5^a^ |
| *hyd-A1 hyd-B1* | 22.6 ± 1.2^a^ | 7.8 ± 0.2^ab^ | 7.8 ± 0.2^ab^ | 23.5 ± 0.7^ab^ |
| *hyd-A2 hyd-B2* | 22.2 ± 0.4^ab^ | 7.7 ± 0.1^ab^ | 7.7 ± 0.1^ab^ | 23.2 ± 0.3^ab^ |
| *hyd-A1 hyd-A2 hyd-B2* | 21.1 ± 1.1^b^ | 7.6 ± 0.2^ab^ | 7.6 ± 0.2^ab^ | 22.8 ± 0.6^b^ |
| *hyd-B1 hyd-A2 hyd-B2* | 22.4 ± 1.7^ab^ | 7.8 ± 0.3^ab^ | 7.8 ± 0.3^ab^ | 23.4 ± 0.9^ab^ |
| *hyd-A1 hyd-B1 hyd-A2* | 22.5 ± 0.6^a^ | 7.8 ± 0.3^ab^ | 7.8 ± 0.3^ab^ | 23.6 ± 0.6^a^ |
| *hyd-A1 hyd-B1 hyd-B2* | 22.5 ± 1.0^ab^ | 7.8 ± 0.2^ab^ | 7.8 ± 0.2^ab^ | 23.4 ± 0.6^ab^ |
| *hyd-A1 hyd-B1 hyd-A2 hyd-B2* | 22.5 ± 0.5^ab^ | 7.8 ± 0.1^a^ | 7.8 ± 0.1^a^ | 23.6 ± 0.3^a^ |
