# Supplementary figures and images for: Genetic dissection of the roles of β-hydroxylases in carotenoid metabolism, photosynthesis, and plant growth in tetraploid wheat (Triticum turgidum L.)
Source: Theor Appl Genet. 2023 Jan 19;136(1):8. doi: 10.1007/s00122-023-04276-3 (PMC9852137; doi:10.1007/s00122-023-04276-3)

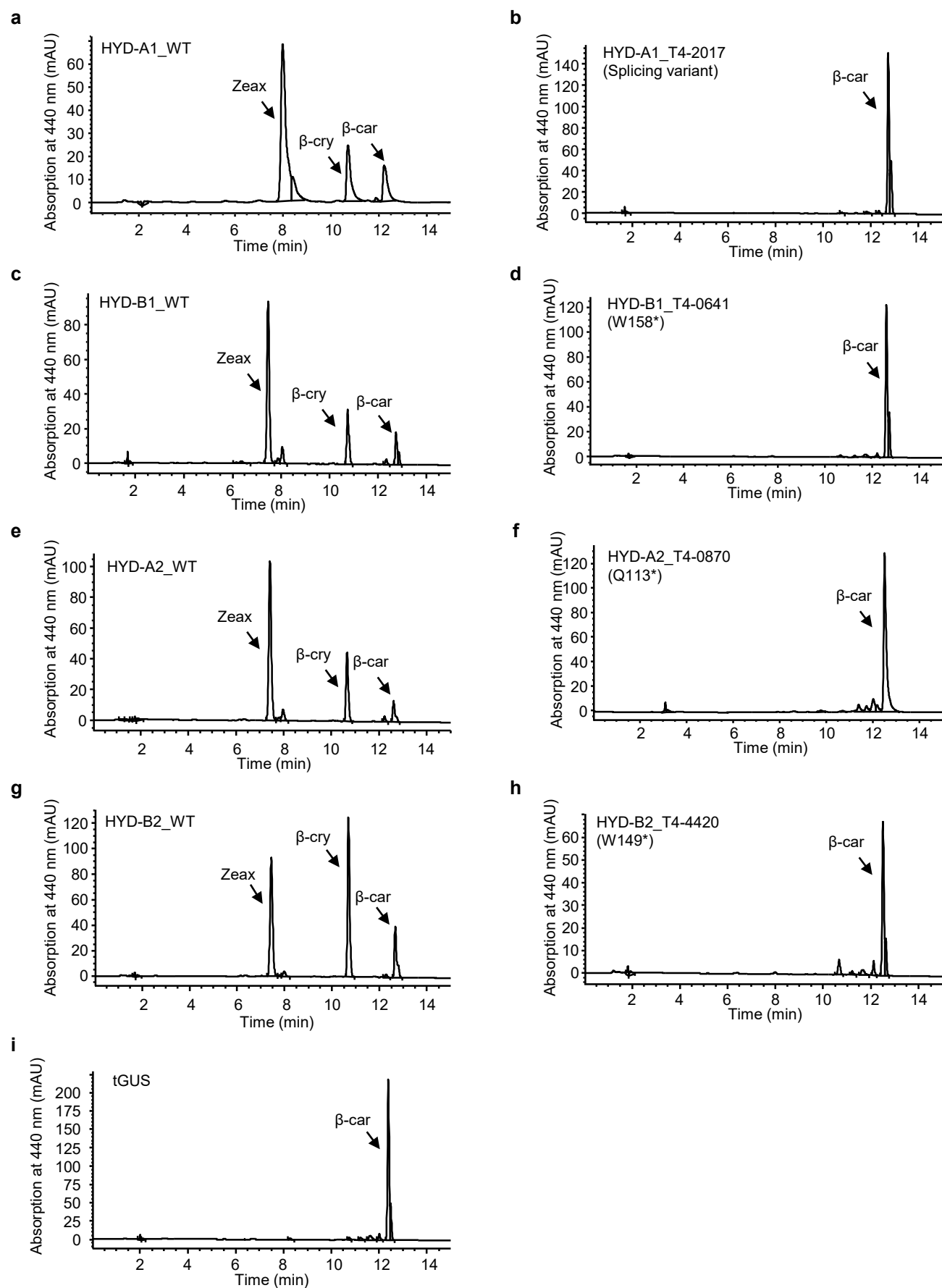

Figure S1

a

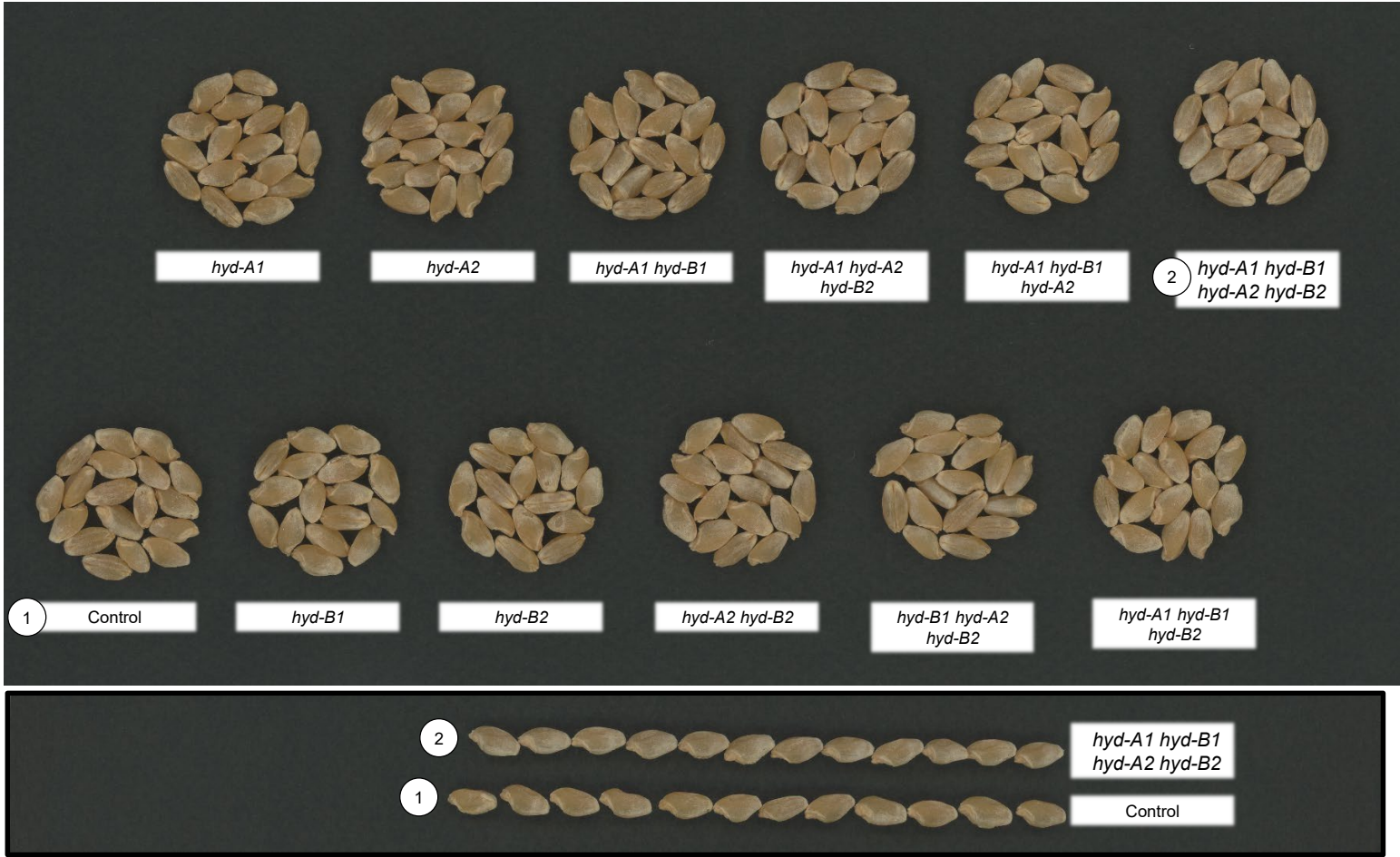

b

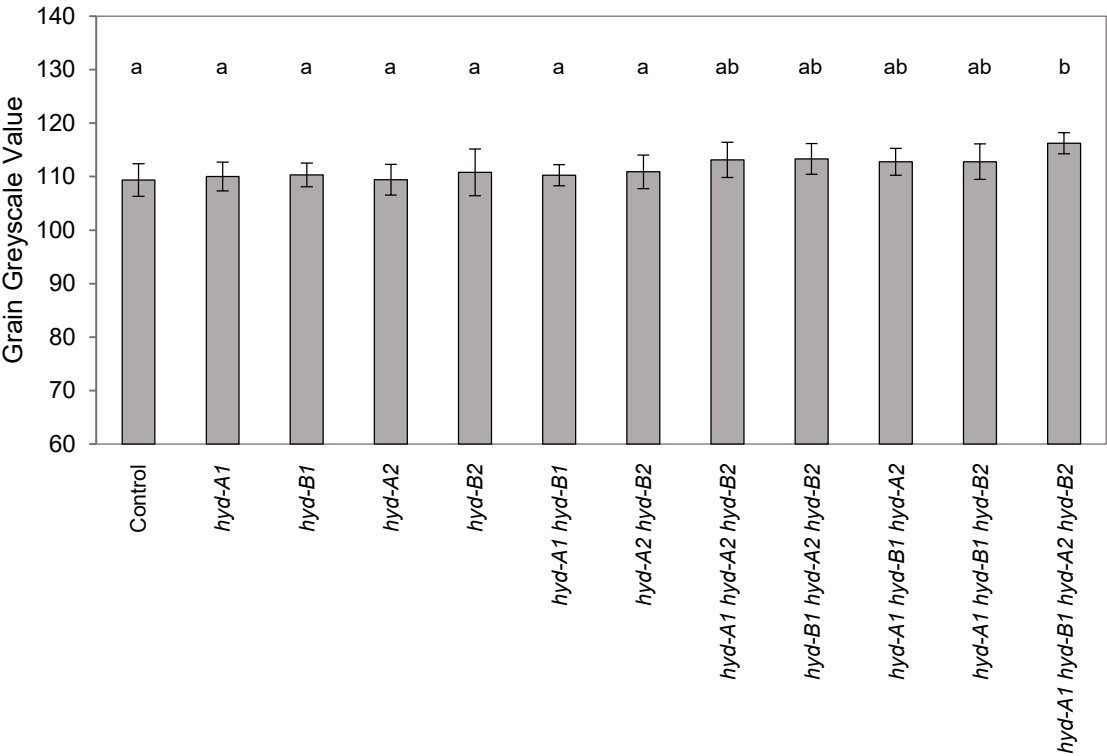

Figure S2

Supplement: Supplementary file 2 — Supplementary file2 (PDF 338 KB) [file 122_2023_4276_MOESM2_ESM.pdf]
